# ALLEVIATING WORRIES OF DEMENTIA AMONG OLDER ADULTS WHO ATTEND AN AGING BRAIN SEMINAR

**DOI:** 10.1093/geroni/igad104.3176

**Published:** 2023-12-21

**Authors:** Grace Simonson, Susan Wehry

**Affiliations:** University of New England College of Osteopathic Medicine, Biddeford, Maine, United States; University of New England College of Osteopathic Medicine, Biddeford, Maine, United States

## Abstract

Dementia worry is prevalent among older adults, and these concerns are often not addressed. Research demonstrates the public lacks accurate knowledge of dementia, which contributes to its negative perception. We hypothesized participation in an aging brain seminar would significantly alleviate dementia worry in older adults. The 45-minute in-person talk covered normal and abnormal aging brain changes, and research-based strategies for healthy brain aging. Attendees completed surveys immediately before and after the seminar. Both surveys included the 12-item Dementia Worry Scale (DWS), which uses Likert scales to assess dementia worry. Two additional Likert questions in the post-survey determined the immediate effect on dementia worry and intent to adopt new strategies. 7 sessions garnered 61 viable pre-post surveys from 160 attendees. 69% reported their dementia worry was somewhat or significantly improved; 68% reported they were very likely or definitely going to take action. Collectively, the Dementia Worry scale was reduced (median pre=19, median post=16, range= 12 - 41), but results were insignificant (p=0.058) likely due to the long-term nature of the questions. The aging brain seminar was successful. It resulted in an immediate reduction of dementia worry and intent to change behavior, and it can be effectively delivered by a medical student. Further research is warranted to determine if the seminar has a long-term effect on dementia worry and sustained adoption of healthy behaviors.

